# DEVELOPMENT OF A FRAILTY INDEX FOR PEOPLE WITH DEMENTIA IN THE HEALTH AND RETIREMENT STUDY

**DOI:** 10.1093/geroni/igac059.1685

**Published:** 2022-12-20

**Authors:** Rachel Wilkie, Jennifer Ailshire

**Affiliations:** University of Southern California, Los Angeles, California, United States; University of Southern California, Los Angeles, California, United States

## Abstract

Frailty indices (FI) have been found to predict adverse outcomes, such as, mortality, hospitalization, and institutionalization in older adults. However, traditional FIs often exclude people with dementia (PWD), who may not be able to consent to or complete all of the standard frailty items. While frailty is a known risk factor for onset of dementia and PWD have higher rates of frailty, little is known regarding how frailty predicts outcomes among PWD. Our study aims to develop an FI for PWD and to examine how this index relates to mortality, hospitalization, and nursing home stays. We used data from the Health and Retirement Study to create a 52-item FI for community-dwelling adults aged 50 years and over classified as having dementia (n = 1,107) in 2014. The index includes deficits in four domains: chronic health conditions, functional status, sensory problems, and overall health and wellbeing. A standardized FI score between 0 and 1 was calculated for each respondent. We used logistic regression to examine associations with FI and 2-year mortality, hospitalization, and nursing home stay, adjusting for age and gender. We found that a 0.1 unit increase in FI was significantly associated with higher odds of 2-year mortality (OR 1.39, p< 0.001), hospitalization (OR 1.45, p< 0.001), and nursing home stay (OR 1.38, p< 0.001) for people with dementia. This study developed an FI which is predictive for adverse outcomes among PWD. Future work should explore how socioeconomic and neighborhood factors contribute to the relationship between frailty and adverse outcomes among PWD.

